# Bridge connection between depression and anxiety symptoms and lifestyles in Chinese residents from a network perspective

**DOI:** 10.3389/fpsyt.2023.1104841

**Published:** 2023-06-15

**Authors:** Shi-Bin Wang, Wen-Qi Xu, Li-Juan Gao, Wen-Yan Tan, Hui-Rong Zheng, Cai-Lan Hou, Fu-Jun Jia

**Affiliations:** ^1^Guangdong Mental Health Center, Guangdong Provincial People's Hospital (Guangdong Academy of Medical Sciences), Southern Medical University, Guangzhou, Guangdong, China; ^2^Nanhai Public Health Hospital of Foshan City, Foshan, Guangdong, China; ^3^Medical College of South China University of Technology, Guangzhou, Guangdong, China; ^4^Department of Psychology, School of Public Health, Southern Medical University, Guangzhou, Guangdong, China

**Keywords:** network, depression, anxiety, lifestyles, population-based study

## Abstract

**Background:**

Lifestyle habits are vital components of the culture of mental health treatment settings. We examined the bridge connection between depressive and anxiety symptoms and lifestyles from a network perspective using a population-based study.

**Methods:**

Face-to-face interviews were conducted with a provincially representative sample of 13,768 inhabitants from the Guangdong Sleep and Psychosomatic Health Survey based on standardized evaluation techniques. We identified the central symptoms by expected influence. The interconnection between depression and anxiety symptoms, as well as the bridge connectivity linking depression–anxiety symptoms and lifestyle factors, were assessed using the bridge centrality index. Network stability and sensibility analyses were performed using a case-dropping bootstrap procedure.

**Results:**

The core symptom that exhibited the highest expected influence was *fatigue or little energy*, followed by *uncontrollable worry, trouble relaxing*, and *sad mood* in the depression-anxiety symptoms network, while *guilt* was the most interconnected symptom and had the highest bridge strength. Surrounding nodes of each node explained an average variance of 57.63%. Additionally, *suicidal thoughts* were recognized as collective bridging symptoms connecting lifestyle variables in the network integrating depression-anxiety symptoms with lifestyle factors. Current tobacco and alcohol consumption were positively associated with *suicidal thoughts* and *irritability*. Habitual diet rhythm and physical exercise frequency were linked to *suicidal thoughts, guilt*, and *poor appetite or overeating*. *Suicidal thoughts, irritability*, and *guilt* indicated the greatest connectivity with lifestyle factors. All networks had high stability and accuracy.

**Conclusion:**

These highlighted core and bridge symptoms could serve as latent targets for the prevention and intervention of comorbid depression and anxiety. It might be crucial for clinical practitioners to design effective and targeted treatment and prevention strategies aiming at specific lifestyles and behaviors.

## Introduction

Mental disorders are prominent public health concerns and the leading causes of global disease burden (1). Depression and anxiety are mental illnesses that affect people all over the world and frequently coexist. The presence of either depressive or anxiety symptoms always increases the risk of developing the other. According to the Netherlands Study of Depression and Anxiety (NESDA), the risk of suffering from a lifetime comorbid anxiety was 0.75 among patients with depressive disorder, while the probability of experiencing depression across the lifespan was 0.81 among those with anxiety (2). Primary prevention has not improved much despite notable advancements in psychotherapy and pharmacotherapy for a range of psychiatric issues in recent years.

In the past years, lifestyle factors were routinely deemed as the general and self-comfort strategies with little clinical correlation (3). However, neglect of lifestyle management tends to cause poor treatment outcomes for psychological problems. Poor diet, inactivity, smoking, and alcoholism continue to be prevalent in the culture of psychological health therapeutic settings. As public health awareness grows, the impact of lifestyles is grabbing more and more attention in preventing and treating psychiatric illness. The Canadian 24-h movement guidelines advocate at least 60 min of moderate exercise and no more than 2 h of sedentary behavior to maintain overall mental health (4). Physical activity, tobacco smoking, and dietary patterns, as well as sleep problems, according to the clinical practice guidelines of mood disorders from the Royal Australian and New Zealand College of Psychiatrists, should be handled first before beginning psychotherapy or pharmacological therapy (5). A systematic meta-review of the high-quality evidence has investigated the causal role of exercise, smoking, diet, and sleep in the onset and prognosis of mental disorders (6). Notwithstanding, a wealth of current evidence (7–10) focused on the influence of a single behavioral factor on mental conditions, such as physical exercise, and typically in connection with depressive disorders (11), while ignoring the comprehensive effect of multiple lifestyle factors on depression and anxiety.

Additionally, earlier research studies were mostly committed to examining the connections between lifestyle factors and symptom total scores or diagnostic categories of one of the illnesses (11, 12). However, these relationships are not only found at a disease level but also at the symptoms level (13), whereas just analyzing the sum of symptom scores or diagnostic categories at the disease level may not be sufficient to identify these interconnections because there are differences in the links between distinct lifestyle factors and various symptoms. Further studies are needed to clarify these specific associations between disease symptoms and lifestyle choices. Recently, an emerging methodology contributed to symptomatology research, indicating different relations connecting individual psychological symptoms with risk factors (14). Network analyses have been extensively applied in the psychopathological field by conceptualizing and visualizing interaction among symptoms over the past few years.

The network approach theories were based on the concept that mental disorders were produced by symptoms that interacted with each other through feedback loops until a self-sustaining state was formed (15, 16). In network models, each symptom was visualized as a node that received stimulus from other mental symptoms and exogenous factors outside the network. Lifestyle factors are examples of external factors that have been introduced into the network of symptoms via bridge connection. Connections between two symptoms or symptoms-factors were visualized as network edges (17). This direct symptoms-factors relation could point to a mutual etiological impact. From the perspective of the core network, the onset of one disorder's symptom may trigger another one as time goes on (18). For example, anhedonia can result in people losing interest in daytime activities and work, which may increase feelings of guilt or sadness later in the day because they did not get all done what they wanted to do. This might provide a theoretical explanation for the comorbidity's underlying mechanisms (19). These core and bridge symptoms are latent targets to prevent and intervene in mental disorders, which is crucial to develop efficient and purposeful treatment strategies for psychiatrists.

Based on a massive sample size of 13,768 residents, this study was designed to examine the role of lifestyle factors in the network of depression and anxiety symptoms. In particular, there were several aims: First, the comorbid network structure of depression and anxiety symptoms would be depicted. Second, the direct connection between depression-anxiety symptoms and different lifestyle clusters would be identified, and the common bridging symptoms were examined. Finally, the networks of varying complexity with or without covariates adjustment would be established, and then the sensitivity and stability of all models were analyzed.

## Methods

### Study population and procedures

The Guangdong Sleep and Psychosomatic Health Survey (GSPHS), a population-based survey with a representative sample of residents aged 18–85 years living in Guangdong province, China, provided the data for this current research. The survey was carried out using a multistage stratified cluster sampling technique between September and November 2019 (20). The sampling procedures in corresponding fine detail have been presented elsewhere (21). The GSPHS team recruited and trained 142 investigators who had at least 2 years of learning experience in the field of medicine and health on standard survey procedures. Participants who satisfied all three of the following requirements were included in this study: (1) ≥ 18 years (born before 1 October 2001); (2) long-standing residents who have lived in Guangdong Province for more than 6 months in a calendar year; and (3) being able to take part in a face-to-face interview. Respondents who were too ill to reply and those who were younger than 18 years were not included in this poll. Each participant gave electronic informed consent before taking the questionnaire at a local neighborhood health center or community clinic. Our investigators conducted in-person interviews with each participant using a series of assessment batteries by online questionnaires. The GSPHS protocol received approval from the Research Ethics Committee of the Guangdong Provincial People's Hospital, Guangdong Academy of Medical Sciences [Reference number: GDREC2018543H (R1)].

### Survey instruments

#### Symptomatology

The 9-item Patient Health Questionnaire (PHQ-9) was applied to measure symptoms of depression over the previous 2 weeks (22). Table 1 illustrates the reference names of the nine items. Each response option was assigned a value of 0 (not at all) to 3 (nearly every day), so the total points of the whole scale ranges from 0 to 27, with higher scores indicating more severe symptoms (23). The satisfactory psychometric qualities for screening depression symptoms have been consistently confirmed in the general population using the Chinese version of PHQ-9 (24). This study's Cronbach's alpha was 0.91, indicating acceptable internal consistency.

**Table 1:** Mean, standard deviation, expected influence, and predictability of depressive and anxiety symptoms assessed using the PHQ-9 and GAD-7.

**Node**	**Items/symptoms**	**M**	**SD**	**Expected influence**	**Predictability**
D1	Anhedonia	0.54	0.69	0.946	0.575
D2	Sad mood	0.49	0.64	1.038	0.622
D3	Trouble sleeping	0.49	0.68	0.752	0.485
D4	Fatigue or little energy	0.59	0.70	1.117	0.630
D5	Poor appetite or overeating	0.42	0.64	0.726	0.451
D6	Guilty	0.42	0.68	0.999	0.584
D7	Trouble concentrating	0.38	0.66	0.848	0.523
D8	Moving slowly or restless	0.28	0.56	0.960	0.547
D9	Suicidal thoughts	0.15	0.43	0.773	0.390
A1	Nervousness	0.46	0.64	0.910	0.605
A2	Uncontrollable worry	0.40	0.66	1.092	0.674
A3	Excessive worry	0.53	0.72	0.989	0.660
A4	Trouble relaxing	0.44	0.68	1.044	0.660
A5	Restlessness	0.56	0.72	1.036	0.630
A6	Irritability	0.30	0.58	0.957	0.600
A7	Feeling afraid	0.31	0.60	0.921	0.584

D1–D9: the nine items of PHQ-9.

A1–A7: the seven items of GAD-7.

M, mean; SD, standard deviation.

The 7-items Generalized Anxiety Disorder (GAD-7) scale was used to assess anxiety symptoms over the 2 weeks (25). The reference name of each item is shown in Table 1. The GAD-7 option settings are identical to those of the PHQ-9 scale, with total scores ranging from 0 to 21, where greater values signify higher levels of anxiety symptoms. The Chinese version of GAD-7 has been identified with excellent retesting reliability and validity in large samples from a primary care setting (26). The Cronbach's alpha coefficient for GAD-7 in our investigation was 0.93.

#### Lifestyles

A collection of questions in the questionnaire served as a lifestyle measurement. For example, participants might have been asked “What kind of habitual dietary rhythm do you have?” and then the answers have been converted into a score relying on the following four classes: 1 = Regular 3 meal/day; 2 = Regular 2 meal/day; 3 = Regular 4 meal/day, and 4 = Irregular diet rhythm. For physical exercise frequency, respondents were investigated with the question “How often do you work out in the past year?” for which, the response was categorized into four levels: 1 = more than 3 times/week; 2 = 1–2 times/week; 3 = 1–3 times/month, and 4 = hardly or never exercise. Tobacco use was classified into never smoked, former smoker, and current smoker. A current smoker was described as smoking at least one cigarette per day and for 6 months or longer. A current drinker in alcohol consumption was designated as consuming on average one time or more of standard alcoholic drinks a week.

### Statistical analysis

R software (version 4.0.5) was applied in all statistical analyses, including network estimation, network comparison and sensitivity analyses, and network accuracy and stability analyses.

#### Network estimation

Different correlations among symptoms, lifestyles, and covariates were estimated from simple to complex models. These models used nodes of networks to denote variables that were linked by edges with varying thickness lines. These edges depended on the strengths of partial correlations between nodes with the confounding effects of all the other nodes being controlled. The skewed distributions of the PHQ-9 and GAD-7 total scores were normalized, and we used non-paranormal transformation to compute non-parametric correlation (27). Considering varying data types in this study, including continuous and categorical variables, mixed graphical models using the function of R package *mgm* (version 1.2-12) were adopted to analyze network predictability (28). The least absolute shrinkage and selection operator (LASSO) in *mgm* contributed to reducing the number of spurious edges and prune network structures (29), which shrank all edge weights and set small weights to zero (30). To avoid the over-fitting issue, the Extended Bayesian Information Criterion (EBIC) was used to select hyperparameter lambda for the strength of the penalty (31). The EBIC was set to 0 or 0.25 to run all models. Expected influence (EI) was designated as an indicator of node importance in this study based on the function *centrality Plot* of the R-package *qgraph* (version 1.6.9) (32). The EI (i.e., node strength) is defined as the normalized sum of all edge weights for a given node and has been proven to be more reliable than the other two centrality indices (i.e., closeness and betweenness) (33, 34). A bridge centrality index (i.e., bridge strength) was also measured based on the function *bridge* of the R-package *networktools* (version 1.4.0) to identify bridge connectivity linking different nodes (35). These nodes in our case are clusters of anxiety and depression symptoms and lifestyles. The absolute sum of node edge weights pointing to another network node is used to calculate the EI of a bridge (36). *R*^2^ was used to estimate the predictability of each node by taking each node in turn and regressing all other nodes on it (37, 38). The network visualizations were conducted using the R package *qgraph* (version 1.6.9) (17). The extent of the correlation was indicated by the thickness of the edge. Green edges represented positive relationships, whereas red edges demonstrated negative relationships.

#### Network comparison and sensitivity analyses

The R-package Network Comparison Test (version 2.2.1) on permutation-based test was used to compare networks among different gender subgroups (39). There were two invariance tests containing network structure invariance and global strength invariance. We evaluated the sensitivity of networks by adding covariates of age and gender. In this study, various variables were modeled using mixed model analysis in *mgm* package, such as continuous variables for scores, nominal variables for gender, and ordinal variables for lifestyle factors.

#### Network accuracy and stability analyses

Network stability of node strength and bridge strength were examined through the case dropping bootstrap procedure via R-package *bootnet* (version 1.5.0) (40). Specifically, we compared centrality indices after reducing cases to the original ones, after randomly removing a subset of samples. When the centrality index did not differ significantly after a large number of samples were excluded from the dataset and the correlation stability coefficient (CS-C) was used to quantify, networks were judged to be robust (40). In general, the CS-C values were typically >0.5 or, better yet, >0.7, indicating a stable network (40). The function *bootnet* was used to visualize the bootstrapped difference tests for edge weights and the results of 95% confidence intervals of all edge weights by 500 bootstraps.

## Results

### Sample characteristics

Out of the 17,132 participants, 13,768 responded to the survey for an overall response rate of 80.4%. The sample's age ranges from 18 to 85 years, with a mean age of 34.3 years (SD = 11.04). A total of 6,159 (44.7%) participants were men and 7,609 (55.3%) were women. There were 10,633 (77.2%) participants who had never smoked tobacco, whereas 2,074 (15.1%) participants were current smokers and 1,061 (7.7%) participants were former smokers; 2,361 (17.1%) people consumed alcohol at least once per week; 11,083 (80.5%) participants had regular three meals per day, 944 (6.9%) had two meals per day, 802 (5.8%) had four meals per day, and 939 (6.8%) had an irregular diet rhythm. There were 3,730 (27.1%) participants who hardly or never exercised, 4,488 (32.6%) occasionally exercised, 2,813 (20.4%) exercised one or two times per week, and 2,737 (19.9%) did frequent exercise (Supplementary Table 1). The mean overall PHQ-9 and GAD-7 scores were 3.76 ± 4.38 and 3.00 ± 3.85, respectively. The descriptive statistics of all symptoms are presented in Table 1.

### Network of depression-anxiety symptoms

**Figure 1:**
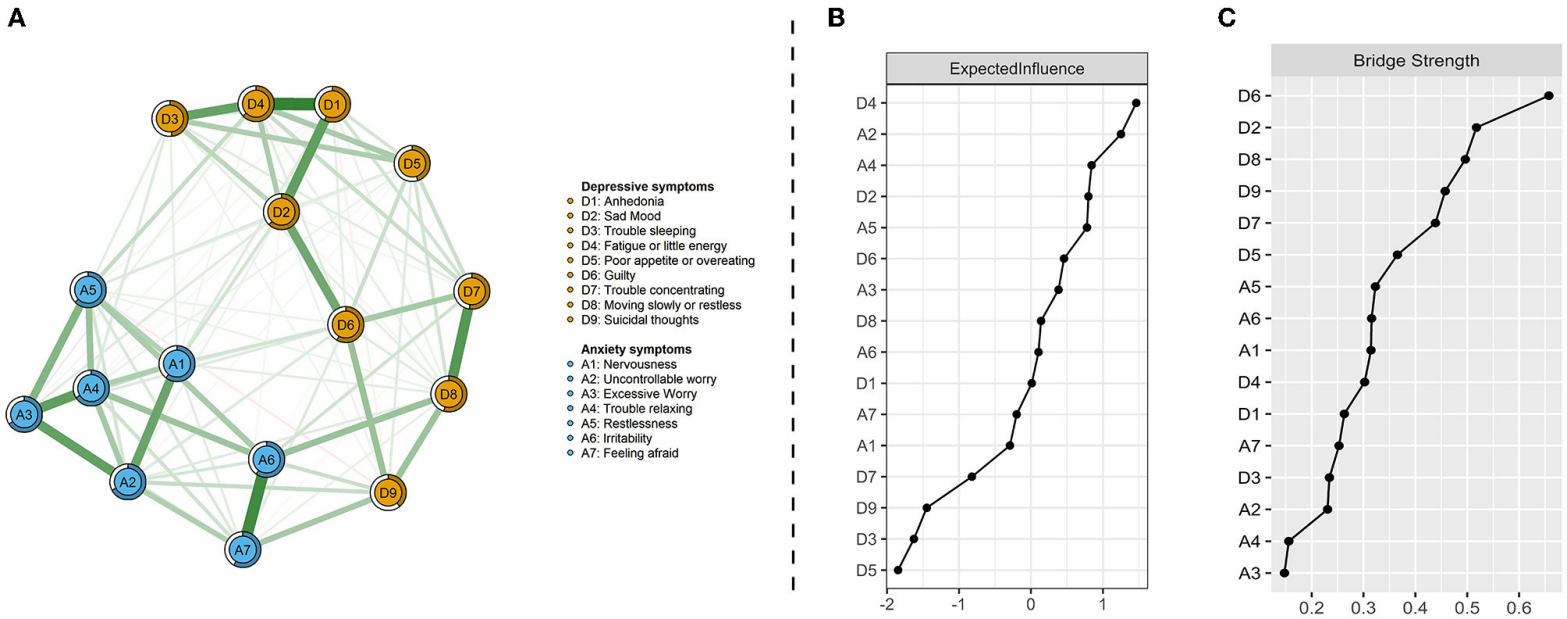
Network visualization of depression and anxiety symptoms **(A)**, centrality index of expected influence **(B)**, and bridge centrality index of bridge strength **(C)**. Green edges indicate positive correlations and red edges indicate negative correlations; the thickness of lines is proportional to the strength of the correlation. The darker outer circle of each node displays predictability. The right panels respectively show the values of expected influence and bridge strength in order.

Figure 1A presents the network of depression and anxiety symptoms without controlling covariates. The corresponding correlative matrix is shown in Supplementary Table 2. For depressive symptoms, the strongest association pertained to the edges between *anhedonia* and *fatigue or little energy* (D1–D4). The relationship between *trouble sleeping* and *fatigue or little energy* (D3–D4), *anhedonia* and *sad mood* (D1–D2), *sad mood* and *guilty* (D2–D6), *trouble concentrating* and *moving slowly or restlessness* (D7–D8), were also strong. For anxiety symptoms, the strongest association pertained to the edges between *irritability* and *feeling afraid* (A6–A7). The relationship between *nervousness* and *uncontrollable worry* (A1–A2), *uncontrollable worry* and *excessive worry* (A2–A3), *excessive worry* and *trouble relaxing* (A3–A4), and *excessive worry* and *restlessness* (A3–A5) were also strong. These correlations are visualized in Supplementary Figure 1. The results of centrality and predictability in the depressive and anxiety network among the participants are shown in Figure 1B and Table 1. Fatigue or little energy (D4) has the highest expected influence (i.e., node strength), followed by uncontrollable worry (A2), trouble relaxing (A4), and sad mood (D2). Surrounding nodes of each node explained 57.63% of the variance. The centrality index of bridge strength exhibited that guilty (D6) indicated the highest bridge strength, followed by sad mood (D2), moving slowly or restlessness (D8), and suicidal thoughts (D9; Figure 1C).

### Network of depression-anxiety symptoms and lifestyles

**Figure 2:**
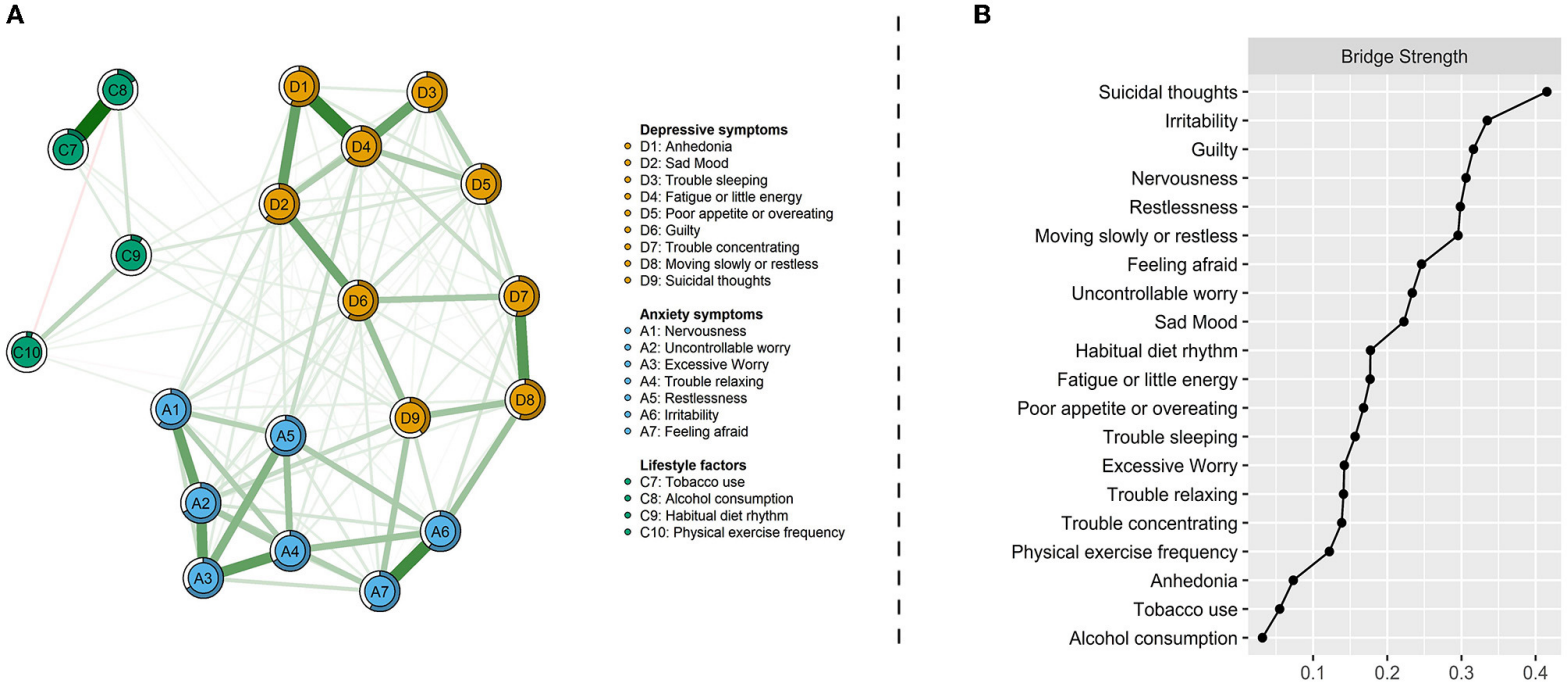
Network visualization of depression-anxiety symptoms and lifestyles **(A)** and bridge centrality index of bridge strength **(B)**. Green edges indicate positive correlations and red edges indicate negative correlations; the thickness of lines is proportional to the strength of the correlation. The darker outer circle of each node displays predictability. Bridge strength represents each node's connectivity with the other network.

Figure 2A displays the relationships between depression-anxiety symptoms and lifestyle factors, and Table 2 lists their direct interconnections. They were mostly positively associated, but only two negative edges were found in a total of 20 interrelating nodes. Both current tobacco and alcohol consumption were positively linked with suicidal thoughts (D9) and irritability (A6). Habitual diet rhythm and physical exercise frequency shared connections with poor appetite or overeating (D5), guilty (D6), and suicidal thoughts (D9). Suicidal thoughts were identified as common bridging symptoms in the network of comorbid depression-anxiety and lifestyles. In addition, a unique relationship between irregular diet rhythm and trouble sleeping (D3), and the respective relationships between reduced physical exercise frequency and fatigue or little energy (D4), and restlessness (A5) were also found (Table 2). This has been confirmed by the bridge centrality index, which indicated that suicidal thoughts, irritability, and guilty exhibited the highest connectivity with lifestyles (Figure 2B).

**Table 2:** Bridge connections between lifestyles and depression-anxiety symptoms.

**Lifestyle factors**	**Bridging depression-anxiety symptoms**	
	**Crude model** ^a^	**Adjusted model** ^b^
Tobacco use	Suicidal thoughts and irritability	Poor appetite or overeating, suicidal thoughts, and irritability
Alcohol consumption	Suicidal thoughts and irritability	Suicidal thoughts
Habitual diet rhythm	Trouble sleeping, poor appetite or overeating, guilt, and suicidal thoughts	Trouble sleeping, poor appetite or overeating, guilty, and suicidal thoughts
Physical exercise frequency	Fatigue or little energy, poor appetite or overeating, guilt, suicidal thoughts, and restlessness	Fatigue or little energy, poor appetite or overeating, guilt, moving slowly, or restlessness

a Crude model is for the network of depression and anxiety symptoms without covariates (i.e., network Figure 1A).

b Adjusted for age and gender.

### Network comparison and sensitivity analyses

When correcting for age and gender, there was no apparent difference in overall network structure and connectivity of depression-anxiety symptoms (Figure 1A vs. Supplementary Figure 2), in which key central and bridge symptoms remained the same. As for the network incorporating lifestyles clusters, changes in bridge connections are demonstrated in Table 2. Compared to that without adjusting age and gender (Figure 2A vs. Supplementary Figure 3), relations between habitual diet rhythm and depression-anxiety symptoms were preserved. Tobacco use increased interconnection with poor appetite or overeating, whereas alcohol consumption cut down the link with irritability. Direct interrelation between physical exercise frequency and suicidal thoughts and restlessness disappeared, while an association with moving slowly or restlessness emerged. All network models were reconstructed with a more conservative lambda of 0.25 (see Supplementary Figures 4–7), which presented identically with original networks (*r* > 0.9 for all correlation coefficients of adjacency matrices). Finally, in the depression-anxiety symptoms model, the results of networks between different gender subgroups were significantly different in overall structure (M = 0.120, *P* < 0.001) and connectivity (S = 0.203, *P* = 0.01). In another model of depression-anxiety symptoms and lifestyles, networks comparison between male and female residents was statistically different in overall structure (M = 0.126, *P* < 0.001) but not different in connectivity (S = 0.023, *P* = 0.84).

### Network accuracy and stability analyses

After bootstrapping 500 times for all models, these networks presented high stability. All results about edge weights significance and strength centrality difference testing, as well as edge weight accuracy, have been visualized in the Supplementary Figures 8–13. The case-dropping bootstrap analyses showed that all correlations were >0.9, manifesting the networks remained stable, where 70% of observations were randomly excluded from the original sample, the estimated correlation of node centrality between the reduced sample and the original sample was 0.9 at least (Supplementary Figures 14, 15).

## Discussion

To the best of our knowledge, this is the largest population-based study employing the network method to explore the interconnections between the symptoms of depression and anxiety as well as between those symptoms and various lifestyles in China. In the present study, a network around interrelated symptoms was established, such as fatigue or little energy, uncontrollable worry, and trouble relaxing. Furthermore, a number of distinct symptom-lifestyle connections have indeed been discovered, such as the correlations between current tobacco/alcohol consumption and suicidal thoughts and irritability; the correlations among habitual diet rhythm, physical exercise frequency and poor appetite or overeating, guilty, and suicidal thoughts. Suicidal thoughts have been recognized as common bridging symptoms in the network of depression, anxiety, and lifestyles. These results offer fresh information about targeted lifestyle-based regimens that can effectively cure individual depression and anxiety symptoms.

We first examined the interrelation between depression and anxiety symptoms in the general population. The majority of the strongest edges in the entire network of depression-anxiety symptoms lay in the depressive community, which is in line with previous research studies (41, 42). However, a study by Bai et al. (43) utilizing the college students population came to a different conclusion. The discrepancy may be the results from various populations and sample methods. This study discovered the strongest association between anhedonia and fatigue or little energy, which was also the second-strongest edge of the depression and anxiety symptoms network in another study on female nursing students (41). Anhedonia and fatigue are characteristic symptoms of psychodiagnostics and usually occur simultaneously, especially in major depressive disorder (MDD) and schizophrenia based on the Diagnostic and Statistical Manual of Mental Disorders (DSM-V) (44). Indeed, some reports suggested that fatigue and anhedonia share relevant or overlapping constructs (45). Interferon-induced inflammation-altered presynaptic striatal dopamine activity might help us to understand the potential collective mechanism of fatigue and anhedonia (46). In addition, fatigue or little energy has the largest expected influence in the overall model, followed by uncontrollable worry and trouble relaxing, demonstrating their substantial involvement in a comorbid depression-anxiety network and possibly targeted symptoms for intervention. Fatigue is prevalent among the population with a prevalence incidence of 5–45% (47). This is likely attributed to a sedentary lifestyle, a lack of physical activity, and poor sleep quality. Anxiety symptoms, such as uncontrollable worry and trouble relaxing, have also a high expected influence, which is in agreement with previous studies on college students (43). In today's fast-paced culture, people's lives have become riddled with anxious moods as a result of life stress, work rhythm, interpersonal relationships, and social difficulties. Relaxation, cognitive behavior therapy, graded exercise therapy, and other therapies could help to alleviate these symptoms. The centrality index of bridge strength is vital to identify influential bridge symptoms that are responsible for exciting and sustaining comorbid psychopathological networks (48). Guilt indicated the highest bridge strength, which was first noticed in the network of depressed mood and co-morbid anxiety states. Guilt is a central feature of depression, which is usually expressed as self-condemned, worthless, powerless, inferiority, hopeless, and helpless emotions (49, 50). Guilt can also contribute to the course of depression. The sad mood has also been observed as a bridge symptom in previous findings on the bridge symptoms in depression and anxiety (36, 41, 42). Additionally, a prior study on the network of female nursing students showed that moving slowly or restlessness was prioritized because of its connection to suicidal thoughts (41). Our findings suggest that targeted therapies that address these bridge symptoms may lower the incidence of co-occurring depression and anxiety.

When lifestyle factors were added into comorbid models, bridge connections between lifestyles and symptoms of depression and anxiety were elucidated. Suicidal thoughts, a serious symptom of depression, were identified as a common bridging symptom between depression-anxiety disorders and lifestyles. Suicidal behavior was portrayed as the outcome of a co-action of mental symptoms, medical conditions, and lifestyle choices (51, 52). The risk of suicide is increased by psychiatric diseases, such as MDD, which have lifestyle behaviors as part of their etiopathogenesis (53, 54). Furthermore, unwholesome lifestyles that contain poor diets, tobacco and alcohol abuse, and a lack of physical activity may enhance the risk of somatic diseases such as obesity, hypertension, metabolic syndrome, and cardiovascular diseases (55). Suicidal thoughts are more common in people suffering from somatic multi-morbidity (56), which is likely due to impairment to the body's system that regulates the stress response, immunologic function, and inflammation (57).

Additionally, the positive connection between current tobacco and alcohol consumption and irritability has also been reported in this study. Emotional disturbance, particularly in depression and anxiety comorbid conditions, has an impact on the construct that regulates aggression, irritability, and agitation, which is an interconnected triad known as “aggression-related behavioral states (ARBS)” (58). Existing preclinical evidence and animal models have shown that nicotinic acetylcholine receptors (nAChRs) affect ARBS (59, 60). Upregulation and desensitization of these receptors may be the cause of smokers' periodic emotional disorders who undergone chronic nicotine exposure (61). A controlled study for alcohol-dependent patients exhibited that drinkers had significantly higher levels of limbic irritability than healthy controls (62). Alcohol consumption is closely connected with the neuropeptides vasopressin (AVP) system in the brain (63). AVP has multiple functions in the cerebrum involving social behaviors, stress, and anxiety responses, and all of these are found to be related to alcohol misuse (64). Alcoholics typically exhibit alcohol cravings, uncontrollable alcohol consumption, and negative mood, including increased anxiety and irritability (65). Irregular diet rhythm was found to be associated with a variety of depressive symptoms including poor appetite or overeating, trouble sleeping, and guilt. There is mounting evidence that depressive disorders have notable lifestyle-driven components (66). Diet and nutrition may significantly alter how depression and anxiety are regulated (67). There are bidirectional relationships between irregular eating habits and poor appetite or overeating. The circadian rhythm is influenced by metabolic processes and energy balance (68). Poor nutrition and feeding schedules might disturb the sleep cycle and thus bring about sleep-related consequences. Physical inactivity was positively connected with fatigue or little energy, poor appetite or overeating, guilt, and restlessness. Long-term and continuous physical activity has been shown to have benefits for emotion management in numerous studies (69). Exercise may induce energy expenditure and heat the body to activate sleep and thus relieve fatigue and increase vigor (70). Physical exercises as one of the treatment strategies for depressive and anxiety disorders involve underlying possible mechanisms relying on the regulation of the hypothalamic-pituitary adrenal axis, upregulation of brain-derived neurotrophic factor, improvement of neurogenesis and cerebral blood flow, and so on (71–73).

The current study outperforms the previous findings in several ways. This is a population-based design that has a large sample size of 13,768 residents and a high response rate of 80.4% using a multistage stratified cluster sampling technique. With consistent results and a conceptual replication of the first presented symptom-lifestyle research, certain shared correlations between individual depression and anxiety symptoms and lifestyle factors were discovered. Notwithstanding, it was important to interpret these results discreetly. First, due to the cross-sectional design, any inferences about the causality were hindered. These associations might be validated by further longitudinal studies and intervention studies. Second, the measurement of depression and anxiety symptoms was performed through self-reported tools, which may introduce reporter bias. Finally, our identification of lifestyle factors was based on straightforward definitions, such as dietary rhythm and exercise frequency. Diet quality and specific patterns, as well as exercise types and intensity, can be explored in future fields.

## Conclusion

Overall, this study was conducted on a big sample size and multiple variables to explore the association between depression and anxiety symptoms and lifestyles. We have not only investigated the comprehensive impact of multiple lifestyle factors but also examined the inner link between lifestyle factors and disease symptoms. Our findings did fill the gap in symptomatology-lifestyle connections. Moreover, the core and bridge symptoms found in this study were latent targets to prevent and intervene in comorbid depression and anxiety. Current research served as a reminder that when depressed and anxious symptoms are reported, clinical practitioners and disease managers should be aware of any potential unhealthy lifestyle concerns that may be present. Healthcare workers should not overlook the influence of personal lifestyles but rather raise the alarm and urge people to seek more precise disease management and follow-up.

## Data availability statement

The raw data supporting the conclusions of this article will be made available by the authors, without undue reservation.

## Ethics statement

The studies involving human participants were reviewed and approved by the Research Ethics Committee of the Guangdong Provincial People's Hospital, Guangdong Academy of Medical Sciences [Reference number: GDREC2018543H (R1)]. The patients/participants provided their written informed consent to participate in this study.

## Author contributions

S-BW conceived the idea of this study, analyzed data, and drafted the manuscript. W-QX had been involved in manuscript original drafting, conceptualization, writing review and editing, and funding acquisition. L-JG assisted in designing the study and advised the data analysis process. W-YT and H-RZ conducted data curation and writing review and editing. C-LH and F-JJ provided critical feedback toward developing the research question and results interpretation. All authors contributed to the article and approved the submitted version.
