# Supplementary figures and images for: Bridge connection between depression and anxiety symptoms and lifestyles in Chinese residents from a network perspective
Source: Front Psychiatry. 2023 Jun 15;14:1104841. doi: 10.3389/fpsyt.2023.1104841 (PMC10308220; doi:10.3389/fpsyt.2023.1104841)

## Network 1a

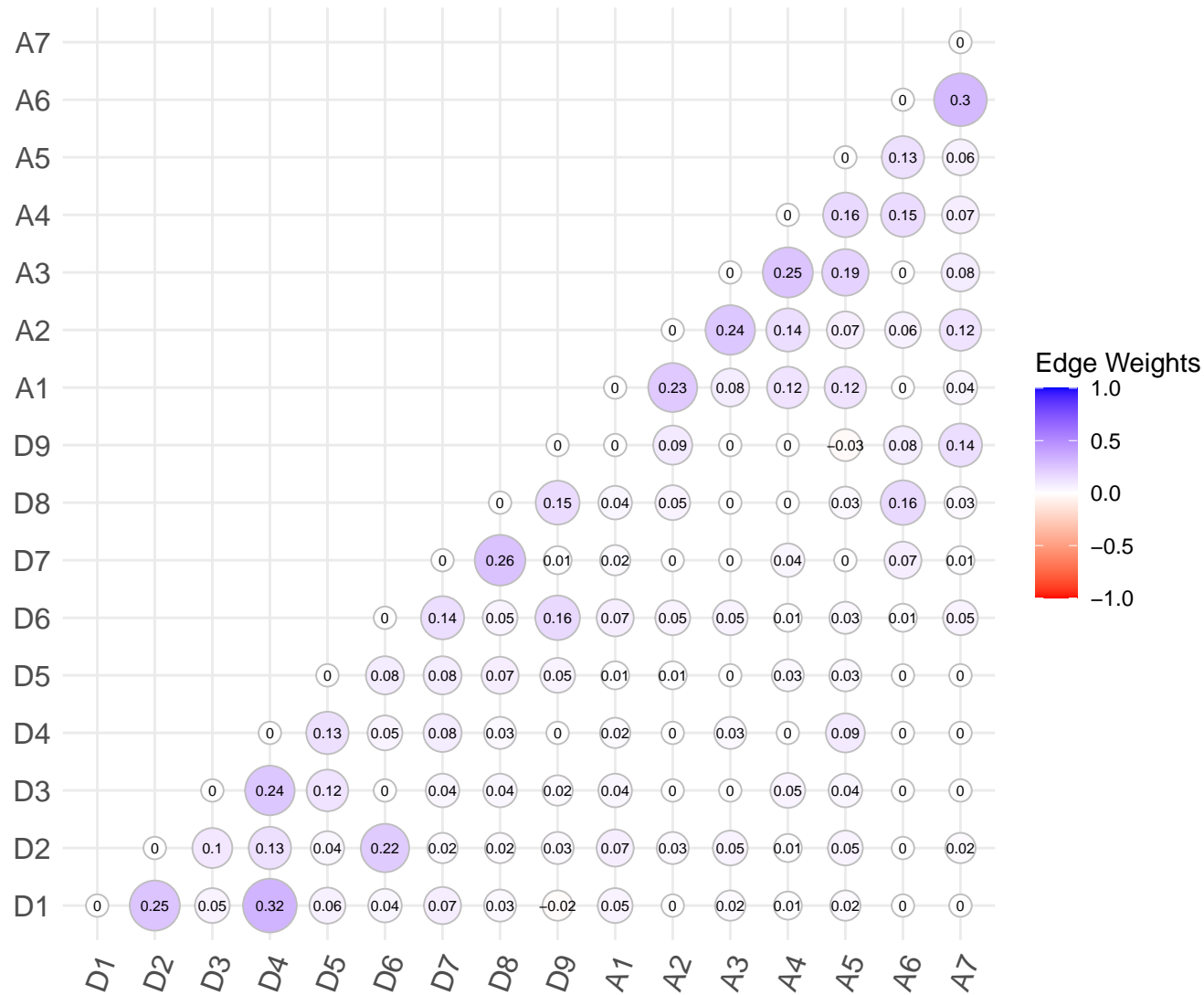

Supplement: Supplementary file 1 [file Data_Sheet_1.PDF]

• Bootstrap mean • Sample

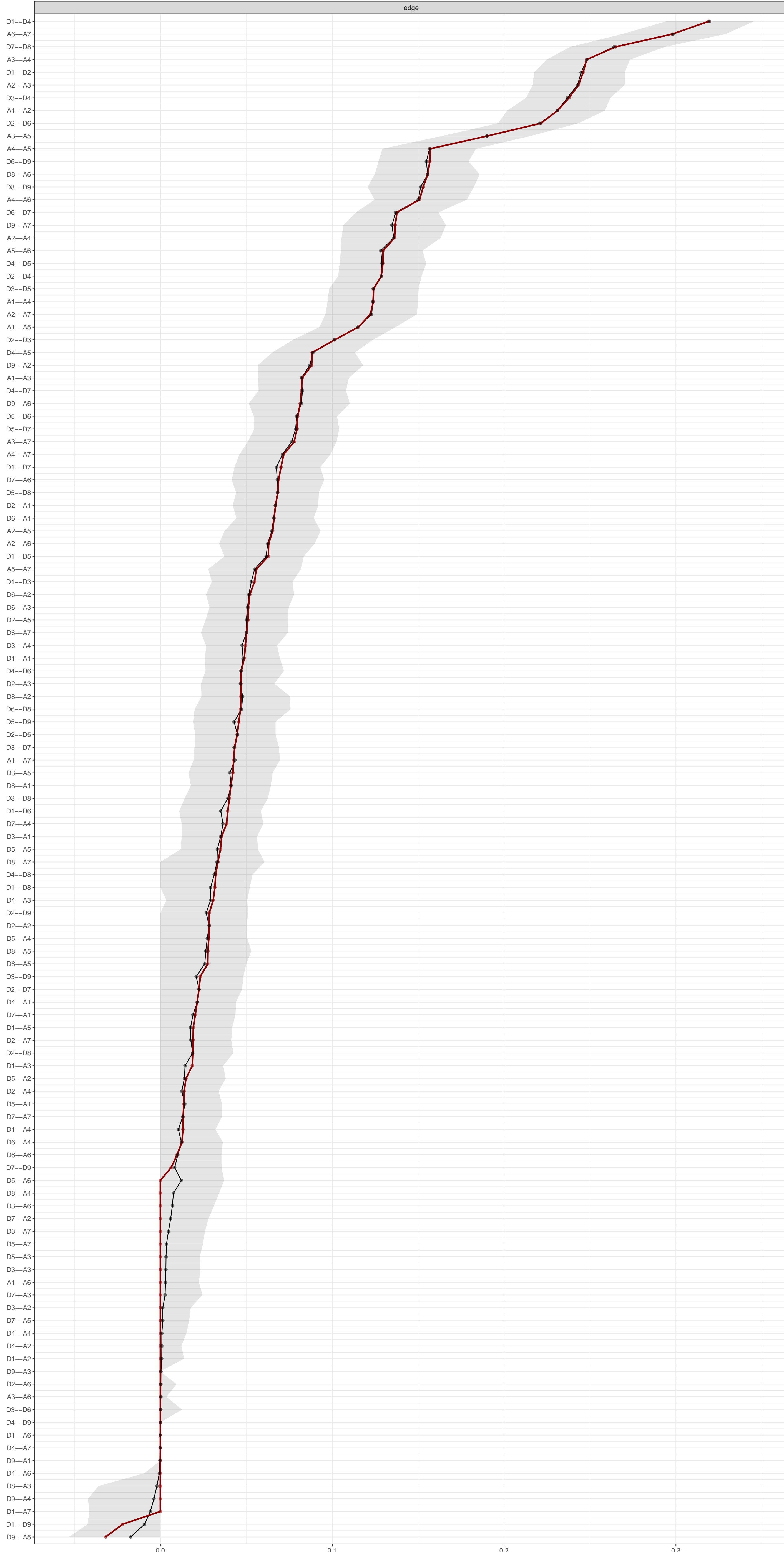

Supplement: Supplementary file 8 [file Data_Sheet_8.PDF]

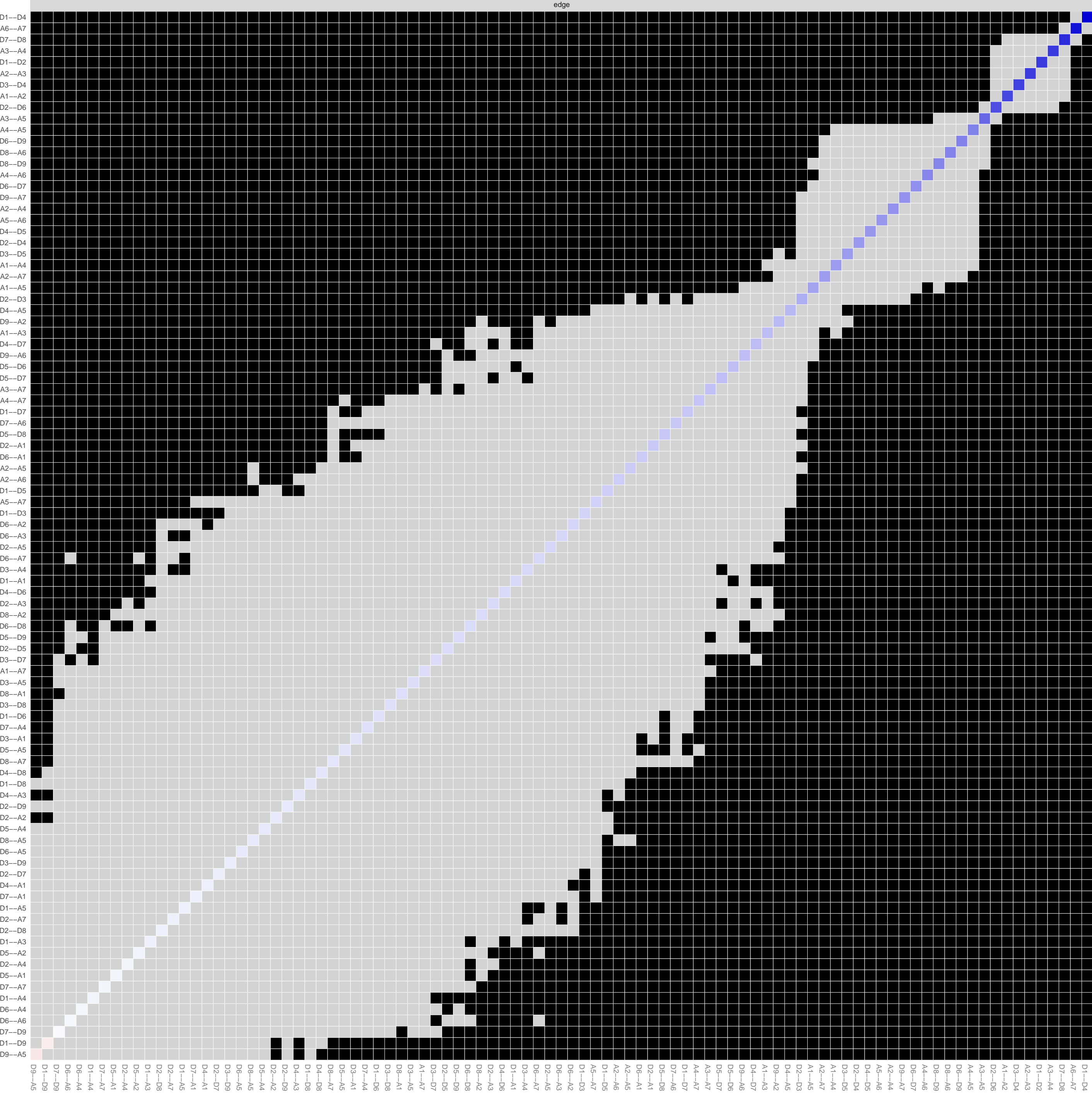

Supplement: Supplementary file 9 [file Data_Sheet_9.PDF]

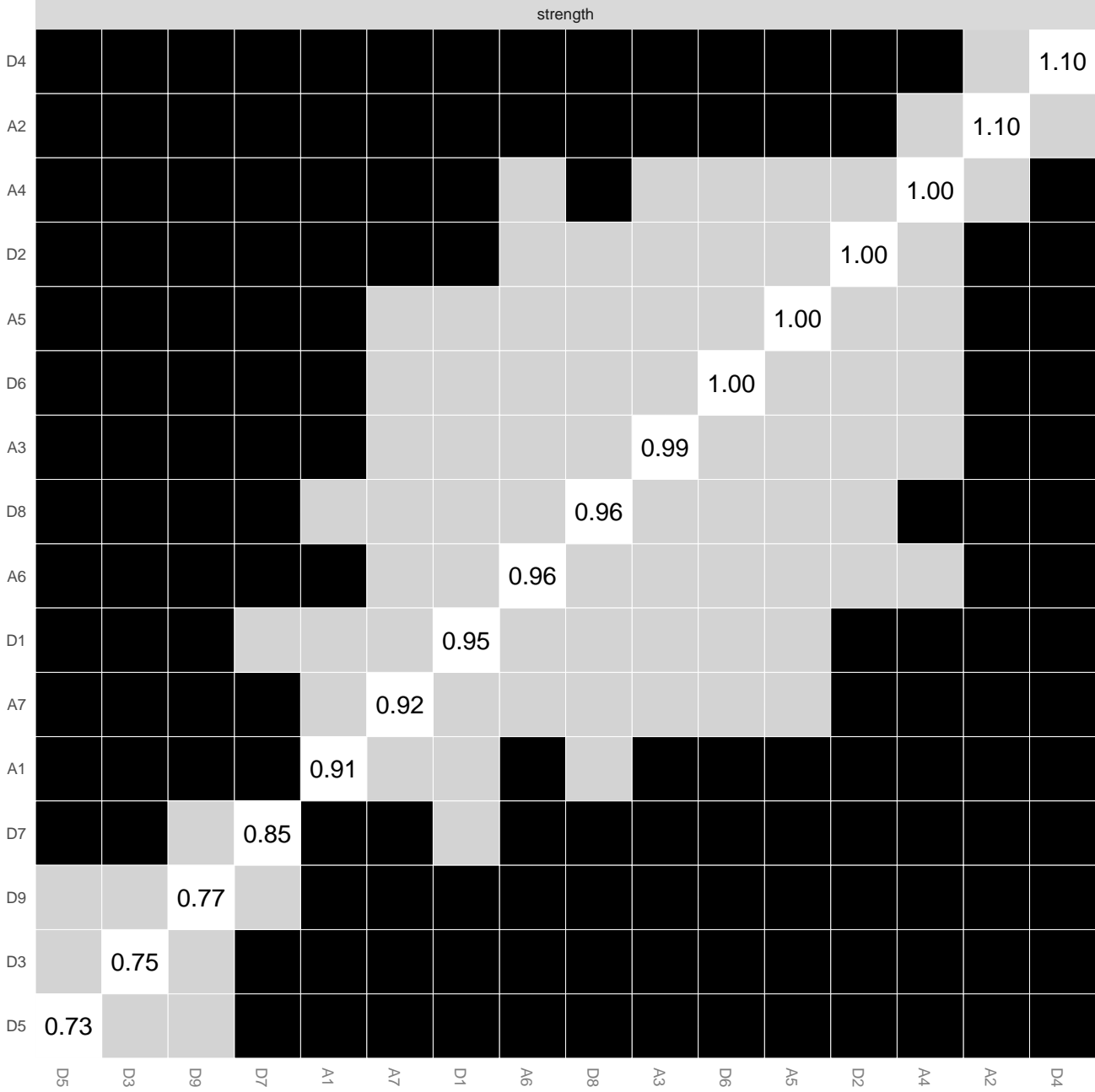

Supplement: Supplementary file 10 [file Data_Sheet_10.PDF]

• Bootstrap mean • Sample

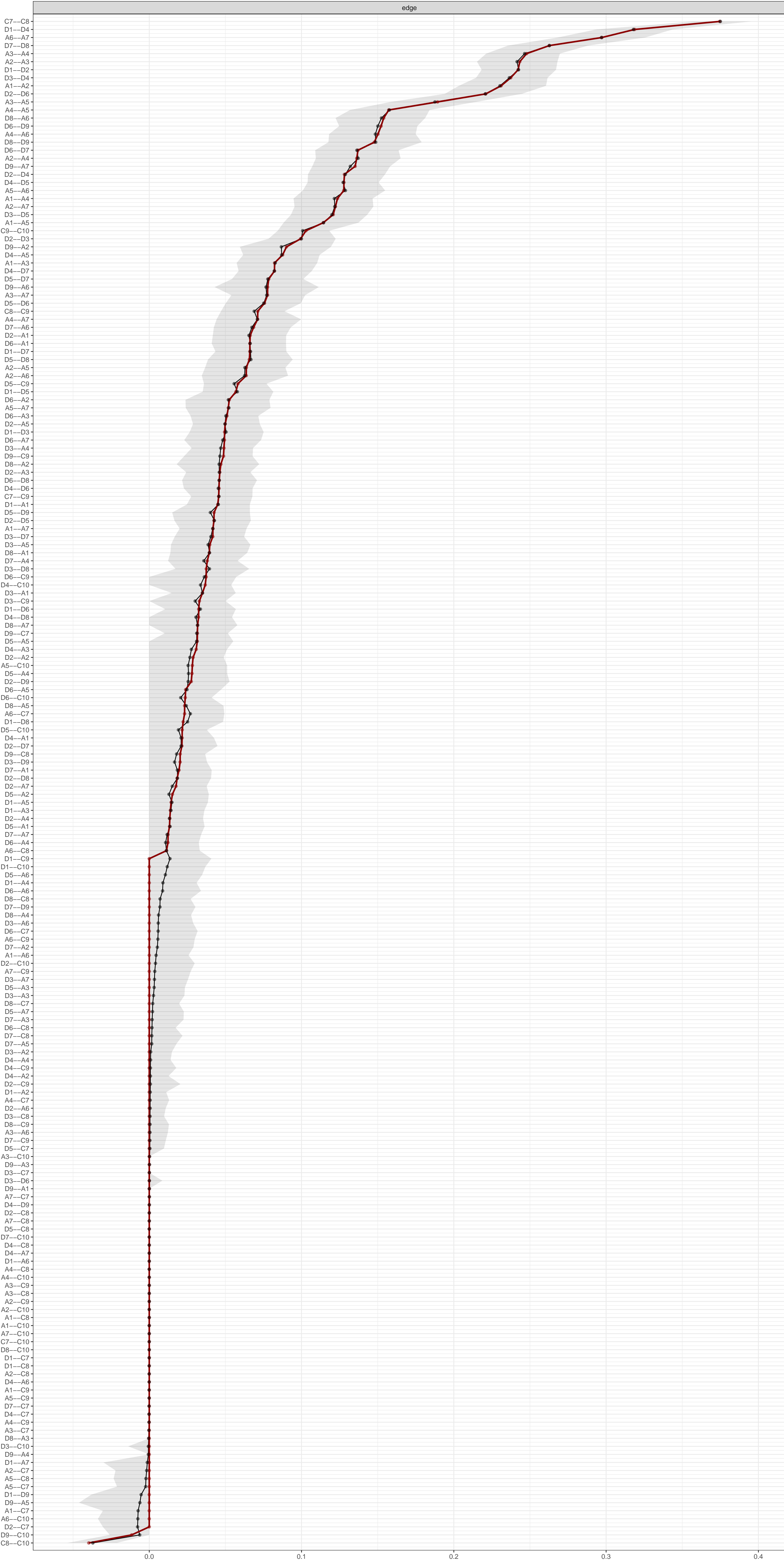

Supplement: Supplementary file 11 [file Data_Sheet_11.PDF]

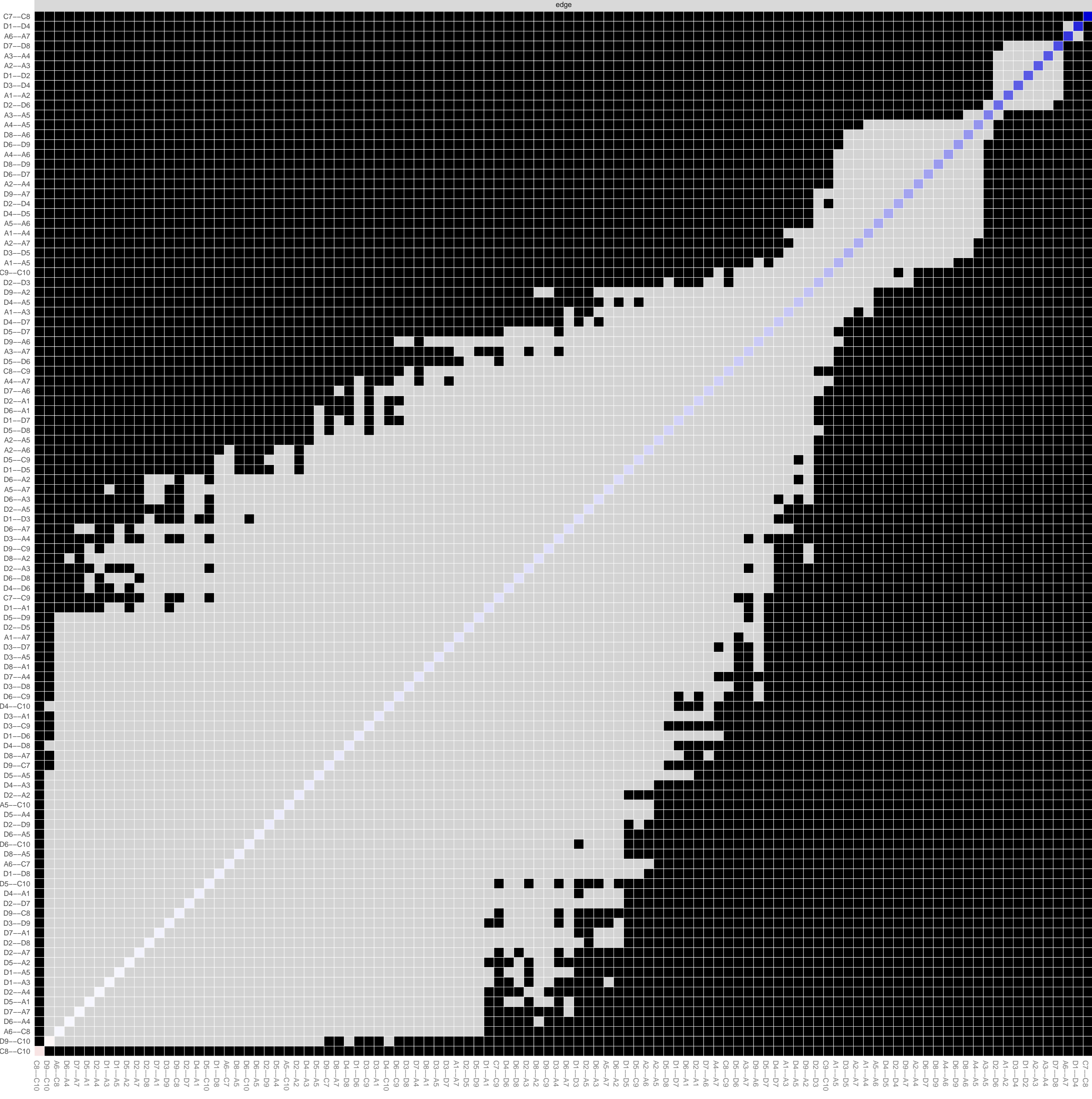

Supplement: Supplementary file 12 [file Data_Sheet_12.PDF]

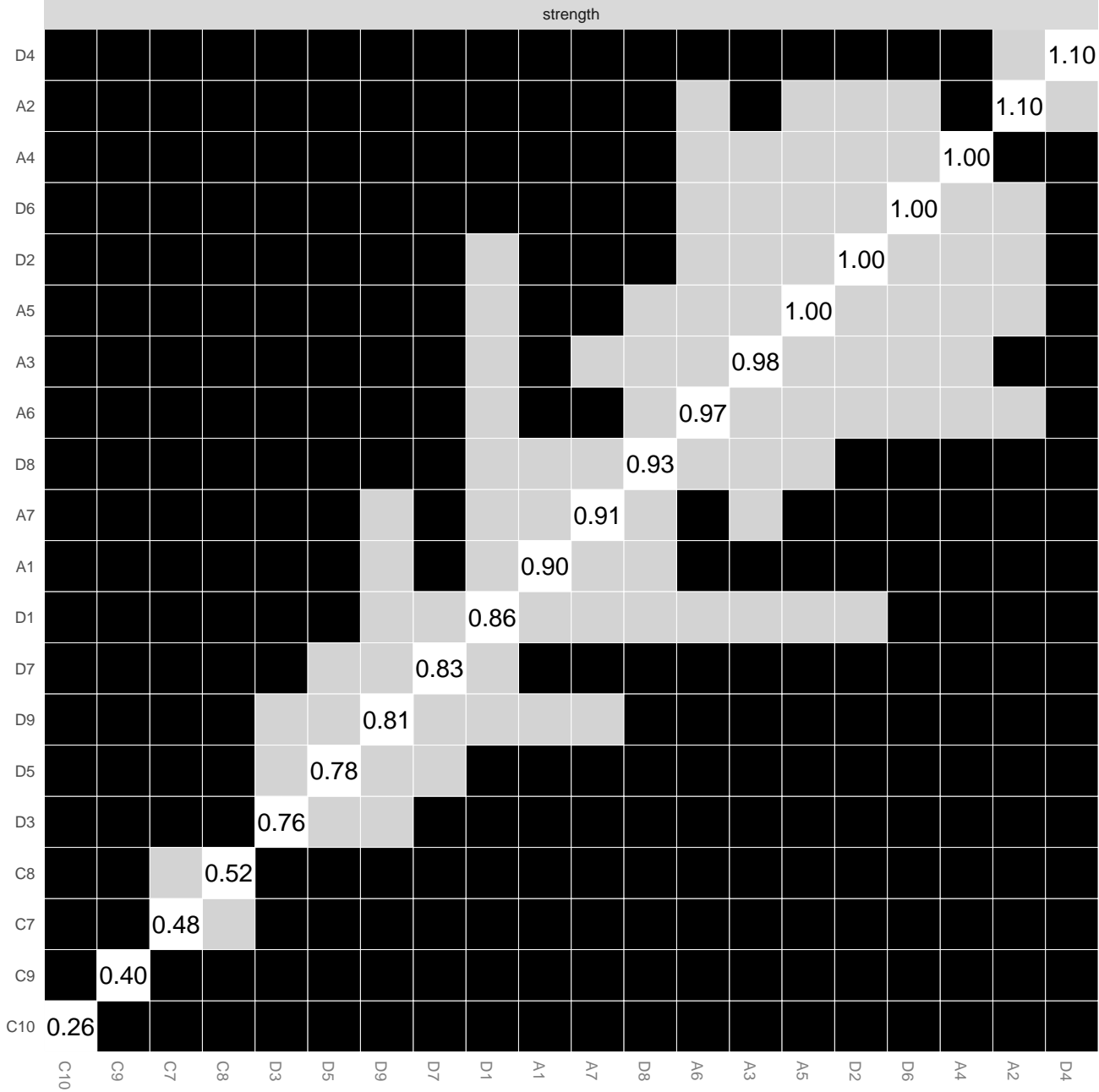

Supplement: Supplementary file 13 [file Data_Sheet_13.PDF]

strength

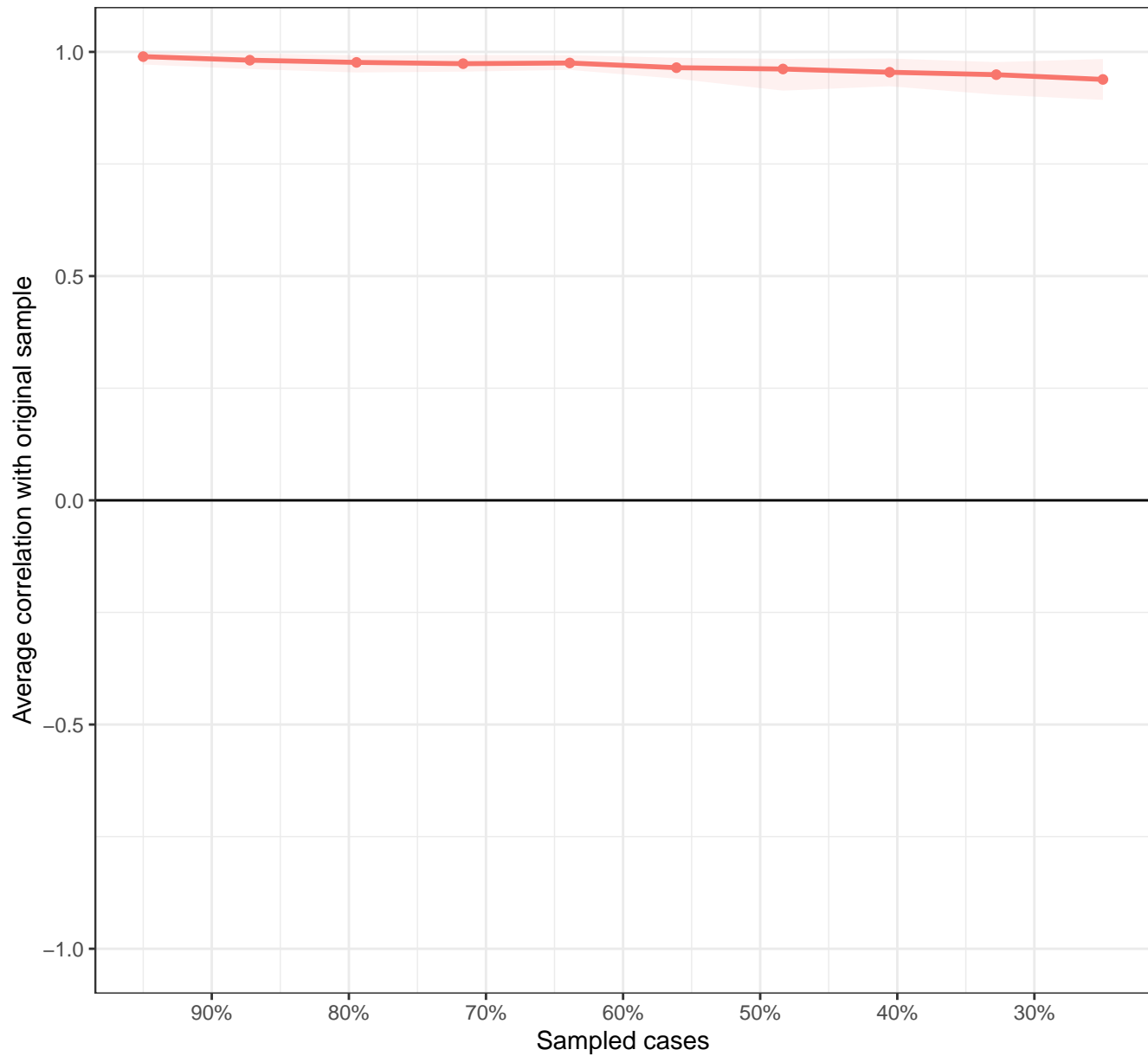

Supplement: Supplementary file 14 [file Data_Sheet_14.PDF]

strength

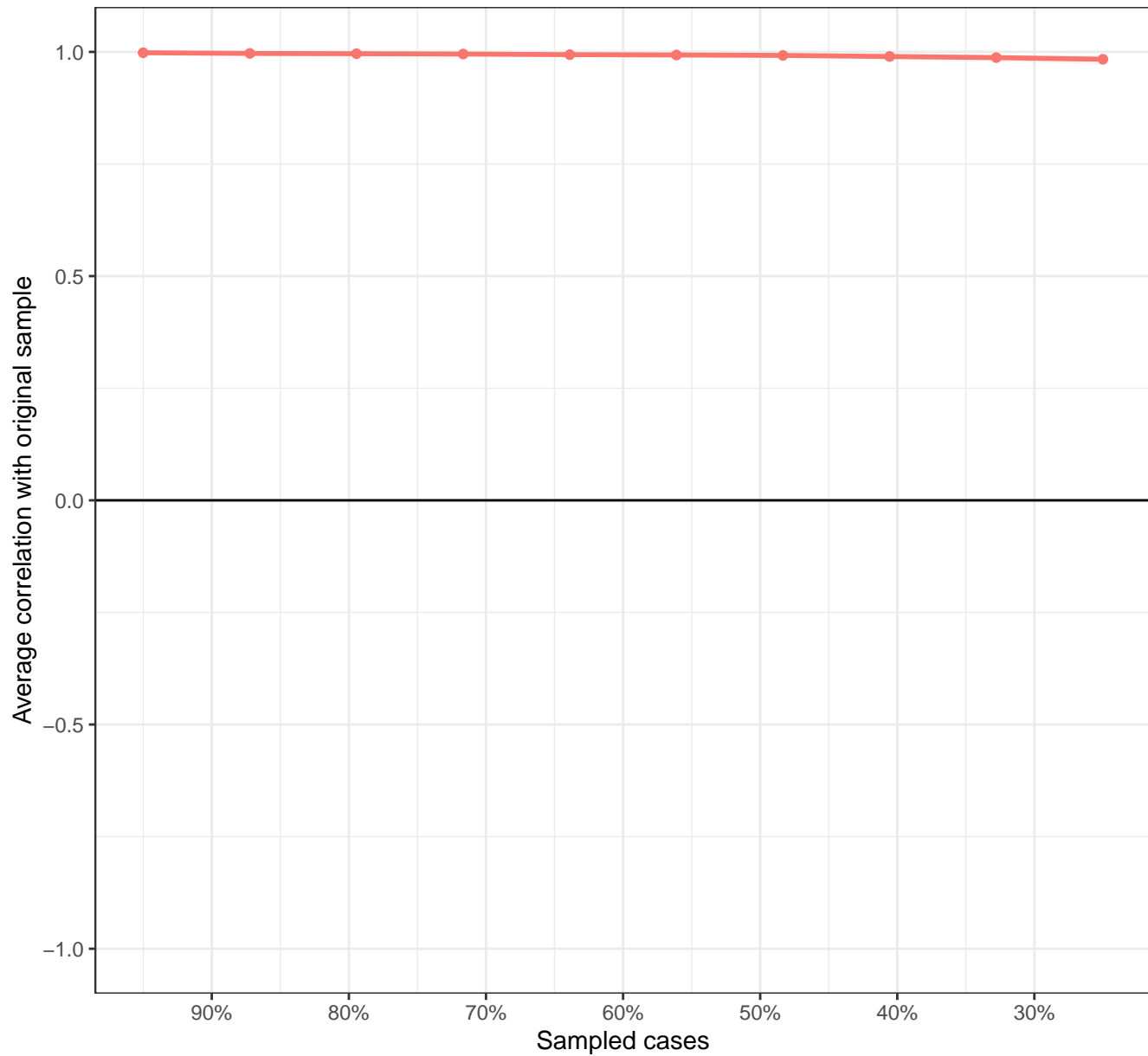

Supplement: Supplementary file 15 [file Data_Sheet_15.PDF]
