# Supplementary material for: Bridge connection between depression and anxiety symptoms and lifestyles in Chinese residents from a network perspective
Source: Front Psychiatry. 2023 Jun 15;14:1104841. doi: 10.3389/fpsyt.2023.1104841 (PMC10308220; doi:10.3389/fpsyt.2023.1104841)
Supplement: Supplementary file 2 [file Data_Sheet_2.PDF]

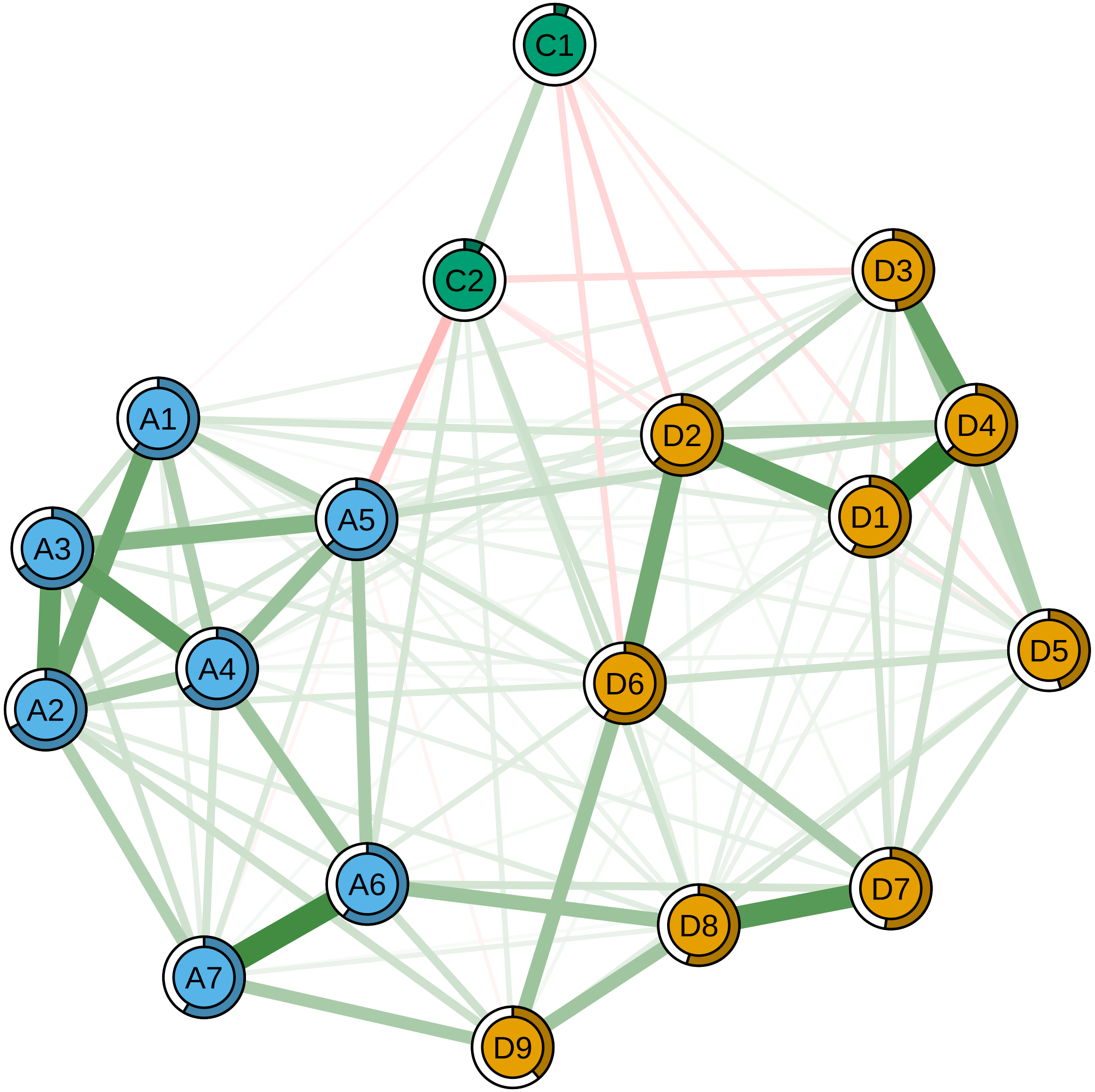

Depressive symptoms

- D1: Anhedonia
- D2: Sad Mood
- D3: Trouble sleeping
- D4: Fatigue or little energy
- D5: Poor appetite or overeating
- D6: Guilty
- D7: Trouble concentrating
- D8: Moving slowly or restless
- D9: Suicidal thoughts

Anxiety symptoms

- A1: Nervousness
- A2: Uncontrollable worry
- A3: Excessive Worry
- A4: Trouble relaxing
- A5: Restlessness
- A6: Irritability
- A7: Feeling afraid

Covariates

- C1: Age
- C2: Gender
