# Supplementary material for: Bridge connection between depression and anxiety symptoms and lifestyles in Chinese residents from a network perspective
Source: Front Psychiatry. 2023 Jun 15;14:1104841. doi: 10.3389/fpsyt.2023.1104841 (PMC10308220; doi:10.3389/fpsyt.2023.1104841)
Supplement: Supplementary file 3 [file Data_Sheet_3.PDF]

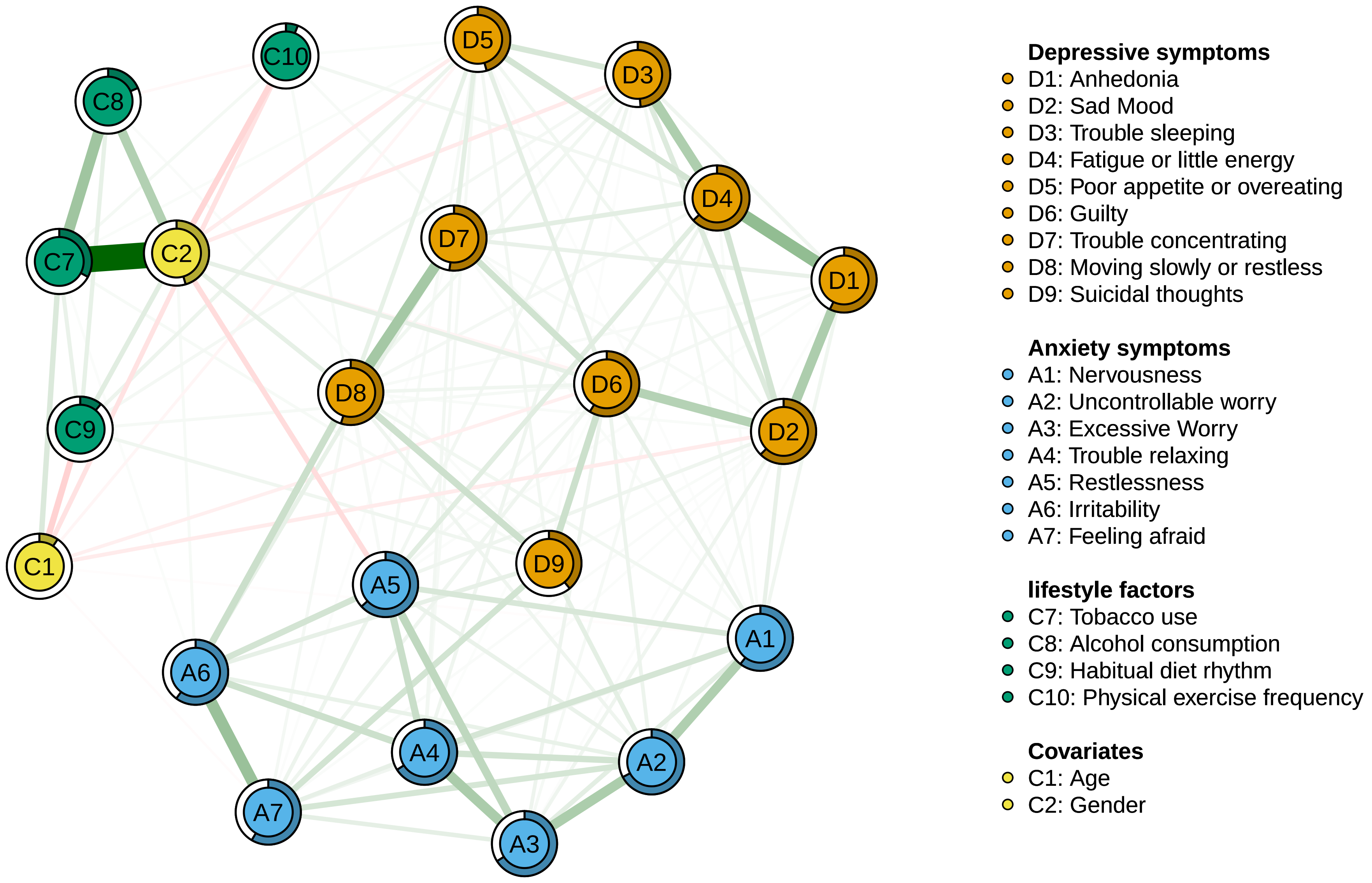
C10
D5
D3
C8
D4
D7
C7
C2
D1
D8
D6
D2
C9
C1
D9
A5
A1
A6
A4
A2
A7
A3
Depressive symptoms
D1: Anhedonia
D2: Sad Mood
D3: Trouble sleeping
D4: Fatigue or little energy
D5: Poor appetite or overeating
D6: Guilty
D7: Trouble concentrating
D8: Moving slowly or restless
D9: Suicidal thoughts
Anxiety symptoms
A1: Nervousness
A2: Uncontrollable worry
A3: Excessive Worry
A4: Trouble relaxing
A5: Restlessness
A6: Irritability
A7: Feeling afraid
lifestyle factors
C7: Tobacco use
C8: Alcohol consumption
C9: Habitual diet rhythm
C10: Physical exercise frequency
Covariates
C1: Age
C2: Gender
