# Supplementary material for: Bridge connection between depression and anxiety symptoms and lifestyles in Chinese residents from a network perspective
Source: Front Psychiatry. 2023 Jun 15;14:1104841. doi: 10.3389/fpsyt.2023.1104841 (PMC10308220; doi:10.3389/fpsyt.2023.1104841)
Supplement: Supplementary file 4 [file Data_Sheet_4.PDF]

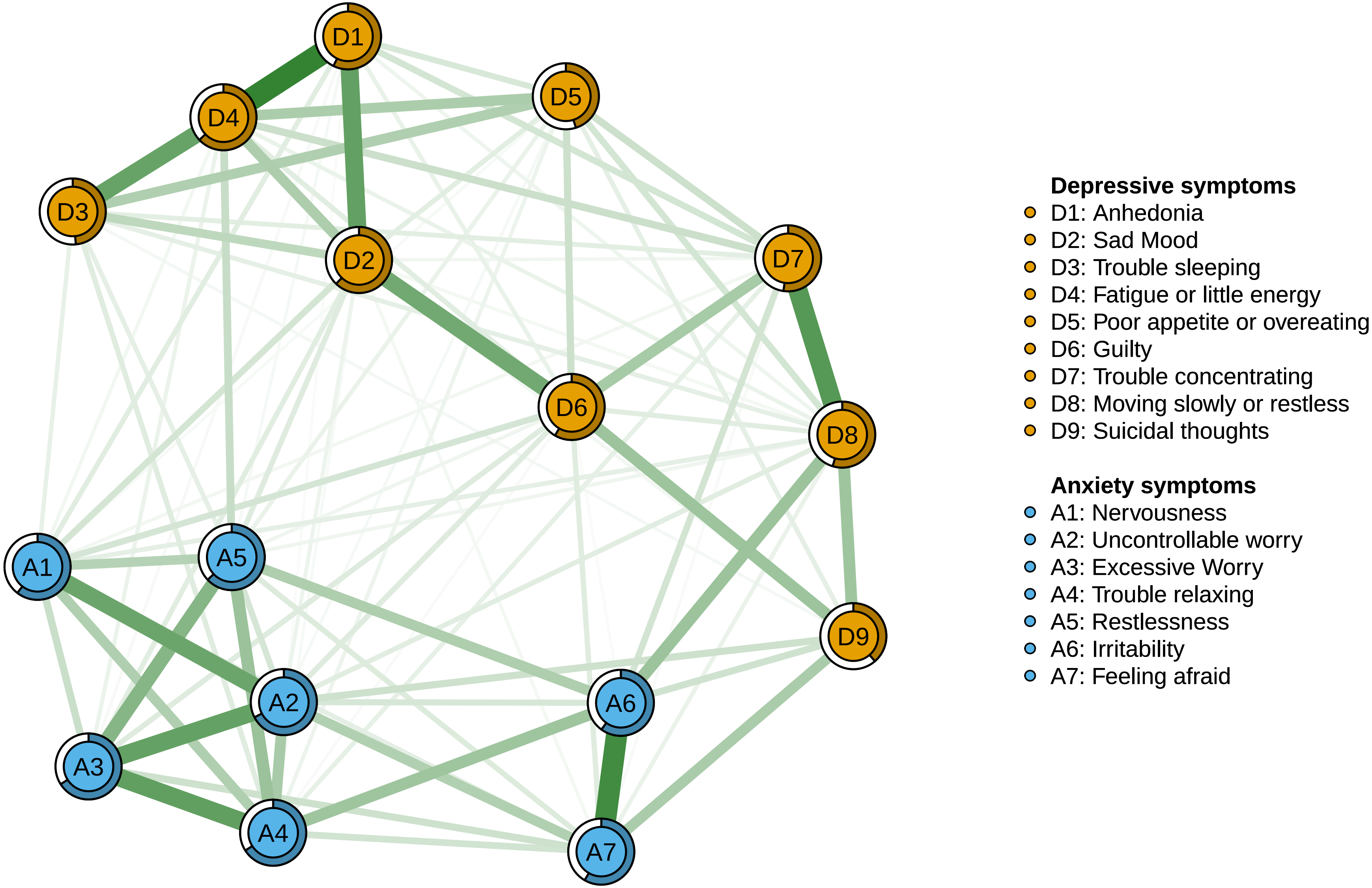
D1
D2
D3
D4
D5
D6
D7
D8
D9
A1
A2
A3
A4
A5
A6
A7
Depressive symptoms
D1: Anhedonia
D2: Sad Mood
D3: Trouble sleeping
D4: Fatigue or little energy
D5: Poor appetite or overeating
D6: Guilty
D7: Trouble concentrating
D8: Moving slowly or restless
D9: Suicidal thoughts
Anxiety symptoms
A1: Nervousness
A2: Uncontrollable worry
A3: Excessive Worry
A4: Trouble relaxing
A5: Restlessness
A6: Irritability
A7: Feeling afraid
