# Supplementary material for: Bridge connection between depression and anxiety symptoms and lifestyles in Chinese residents from a network perspective
Source: Front Psychiatry. 2023 Jun 15;14:1104841. doi: 10.3389/fpsyt.2023.1104841 (PMC10308220; doi:10.3389/fpsyt.2023.1104841)
Supplement: Supplementary file 16 [file Table_1.DOCX]

Supplementary Material

# Bridge connection between depression and anxiety symptoms and lifestyles in Chinese residents from a network perspective

| Items | Content |
| --- | --- |
| Table S1 | Descriptive statistics of the sample(N = 13,768). |
| Table S2 | Spearman correlation matrix between depressive and anxiety symptoms. |
| Figure S1 | Correlation coefficients of network between depressive and anxiety symptoms. |
| Figure S2 | Network structure of depression-anxiety symptoms adjusted for age and gender. |
| Figure S3 | Network structure of depression-anxiety symptoms and lifestyles adjusted for age and gender. |
| Figure S4 | Network structure of depression-anxiety symptoms with a more conservative lambda of 0.25. |
| Figure S5 | Network structure of depression-anxiety symptoms and lifestyles with a more conservative lambda of 0.25. |
| Figure S6 | Network structure of depression-anxiety symptoms adjusted for age and gender with a more conservative lambda of 0.25. |
| Figure S7 | Network structure of depression-anxiety symptoms and lifestyles adjusted for age and gender with a more conservative lambda of 0.25. |
| Figure S8 | Results of the edge weight accuracy using 500 bootstraps for the network of depression-anxiety symptoms. |
| Figure S9 | Bootstrapped results of edge weights significance difference testing for the network of depression-anxiety symptoms . |
| Figure S10 | Bootstrapped results of strength centrality difference testing for the network of depression-anxiety symptoms. |
| Figure S11 | Results of the edge weight accuracy using 500 bootstraps for the network of depression-anxiety symptoms and lifestyles. |
| Figure S12 | Bootstrapped results of edge weights significance difference testing for the network of depression-anxiety symptoms and lifestyles . |
| Figure S13 | Bootstrapped results of strength centrality difference testing for the network of depression-anxiety symptoms and lifestyles. |
| Figure S14 | Stability of centrality index(strength) by case-dropping subset bootstrap for the network of depression-anxiety symptoms . |
| Figure S15 | Stability of centrality index(strength) by case-dropping subset bootstrap for the network of depression-anxiety symptoms and lifestyles . |

Table S1 Descriptive statistics of the sample(N = 13,768).

| Variables | Groups | M (SD) / N (%) |
| --- | --- | --- |
| Age(years) |  | 34.30(11.04) |
| Gender | Male | 6159(44.7%) |
|  | Female | 7609(55.3%) |
| Tobacco use | Never smoking | 10633(77.2%) |
|  | Former smoker | 1061(7.7%) |
|  | Current smoker | 2074(15.1%) |
| Alcohol consumption | < 1 time/week | 11407(82.9%) |
|  | ≥ 1 time/ week | 2361(17.1%) |
| Habitual diet rhythm | Regular 3 meal/day | 11083(80.5%) |
|  | Regular 2 meal/day | 944(6.9%) |
|  | Regular 4 meal/day | 802(5.8%) |
|  | Irregular | 939(6.8%) |
| Physical exercise frequency | ≥ 3 times/week | 2737(19.9%) |
|  | 1-2 times/week | 2813(20.4%) |
|  | 1-3 times/month | 4488(32.6%) |
|  | < once/ month | 3730(27.1%) |
| PHQ-9 total score |  | 3.76(4.38) |
| GAD-7 total score |  | 3.00(3.85) |

Table S2. Spearman correlation matrix between depressive and anxiety symptoms.

|  | D1 | D2 | D3 | D4 | D5 | D6 | D7 | D8 | D9 | A1 | A2 | A3 | A4 | A5 | A6 | A7 |
| --- | --- | --- | --- | --- | --- | --- | --- | --- | --- | --- | --- | --- | --- | --- | --- | --- |
| D1 |  | 0.648 | 0.538 | 0.675 | 0.518 | 0.544 | 0.517 | 0.478 | 0.336 | 0.538 | 0.508 | 0.525 | 0.519 | 0.548 | 0.458 | 0.445 |
| D2 | 0.648 |  | 0.549 | 0.634 | 0.524 | 0.640 | 0.532 | 0.512 | 0.405 | 0.584 | 0.574 | 0.581 | 0.570 | 0.589 | 0.505 | 0.518 |
| D3 | 0.538 | 0.549 |  | 0.613 | 0.509 | 0.487 | 0.483 | 0.467 | 0.346 | 0.494 | 0.481 | 0.497 | 0.511 | 0.519 | 0.452 | 0.440 |
| D4 | 0.675 | 0.634 | 0.613 |  | 0.558 | 0.556 | 0.533 | 0.494 | 0.354 | 0.548 | 0.524 | 0.556 | 0.542 | 0.586 | 0.468 | 0.460 |
| D5 | 0.518 | 0.524 | 0.509 | 0.558 |  | 0.513 | 0.503 | 0.483 | 0.373 | 0.479 | 0.482 | 0.482 | 0.491 | 0.498 | 0.463 | 0.444 |
| D6 | 0.544 | 0.640 | 0.487 | 0.556 | 0.513 |  | 0.559 | 0.539 | 0.474 | 0.568 | 0.583 | 0.569 | 0.562 | 0.563 | 0.533 | 0.536 |
| D7 | 0.517 | 0.532 | 0.483 | 0.533 | 0.503 | 0.559 |  | 0.607 | 0.405 | 0.505 | 0.516 | 0.504 | 0.531 | 0.509 | 0.528 | 0.497 |
| D8 | 0.478 | 0.512 | 0.467 | 0.494 | 0.483 | 0.539 | 0.607 |  | 0.471 | 0.511 | 0.538 | 0.492 | 0.527 | 0.517 | 0.579 | 0.526 |
| D9 | 0.336 | 0.405 | 0.346 | 0.354 | 0.373 | 0.474 | 0.405 | 0.471 |  | 0.388 | 0.459 | 0.398 | 0.410 | 0.382 | 0.452 | 0.469 |
| A1 | 0.538 | 0.584 | 0.494 | 0.548 | 0.479 | 0.568 | 0.505 | 0.511 | 0.388 |  | 0.672 | 0.650 | 0.645 | 0.642 | 0.555 | 0.563 |
| A2 | 0.508 | 0.574 | 0.481 | 0.524 | 0.482 | 0.583 | 0.516 | 0.538 | 0.459 | 0.672 |  | 0.687 | 0.682 | 0.635 | 0.613 | 0.626 |
| A3 | 0.525 | 0.581 | 0.497 | 0.556 | 0.482 | 0.569 | 0.504 | 0.492 | 0.398 | 0.650 | 0.687 |  | 0.701 | 0.675 | 0.572 | 0.587 |
| A4 | 0.519 | 0.570 | 0.511 | 0.542 | 0.491 | 0.562 | 0.531 | 0.527 | 0.410 | 0.645 | 0.682 | 0.701 |  | 0.673 | 0.633 | 0.610 |
| A5 | 0.548 | 0.589 | 0.519 | 0.586 | 0.498 | 0.563 | 0.509 | 0.517 | 0.382 | 0.642 | 0.635 | 0.675 | 0.673 |  | 0.601 | 0.570 |
| A6 | 0.458 | 0.505 | 0.452 | 0.468 | 0.463 | 0.533 | 0.528 | 0.579 | 0.452 | 0.555 | 0.613 | 0.572 | 0.633 | 0.601 |  | 0.662 |
| A7 | 0.445 | 0.518 | 0.440 | 0.460 | 0.444 | 0.536 | 0.497 | 0.526 | 0.469 | 0.563 | 0.626 | 0.587 | 0.610 | 0.570 | 0.662 |  |
| Note: GAD, Generalized Anxiety Disorder; PHQ, Patient Health Questionnaire; D1: Anhedonia, D2: Sad Mood, D3: Trouble sleeping, D4: Fatigue or little energy, D5: Poor appetite or overeating, D6: Guilty, D7: Trouble concentrating, D8: Moving slowly or restless, D9: Suicidal thoughts; A1: Nervousness, A2: Uncontrollable worry, A3: Excessive worry, A4: Trouble relaxing, A5: Restlessness, A6: Irritability, A7: Feeling afraid. | | | | | | | | | | | | | | | | |
